# Angiogenesis genotyping in the selection of first-line treatment with either sunitinib or pazopanib for advanced renal cell carcinoma

**DOI:** 10.18632/oncotarget.9229

**Published:** 2016-05-09

**Authors:** Maristella Bianconi, Luca Faloppi, Cristian Loretelli, Antonio Zizzi, Riccardo Giampieri, Alessandro Bittoni, Kalliopi Andrikou, Michela Del Prete, Luciano Burattini, Rodolfo Montironi, Mario Scartozzi, Stefano Cascinu

**Affiliations:** ^1^ Department of Oncology, AOU Ospedali Riuniti, Polytechnic University of The Marche Region, Ancona, Italy; ^2^ Institute of Pathological Anatomy, AOU Ospedali Riuniti, Polytechnic University of The Marche Region, Ancona, Italy; ^3^ Department of Medical Oncology, Università degli Studi di Cagliari - Azienda Ospedaliero Universitaria, Cagliari, Italy

**Keywords:** renal cell carcinoma, VEGF, angiogenesis, sunitinib, pazopanib

## Abstract

**Introduction:**

Recent data from the COMPARZ study seem to suggest a non-inferiority of pazopanib confronted with sunitinib in PFS and OS. We previously reported how VEGF and VEGFR polymorphisms might have a predictive role in patients treated with first-line sunitinib. Aim of our study was to investigate whether tumour angiogenesis genotyping could influence clinical outcome in RCC patients treated with either sunitinib or pazopanib, in order to help clinicians select the appropriate treatment for each patient.

**Results:**

19 patients were treated with pazopanib while 78 received sunitinib. VEGF A rs833061 resulted significant in PFS in sunitinib vs pazopanib patients (CC+CT>TT in sunitinib, TT>CC+CT in pazopanib; p<0,0001); VEGF A rs2010963 resulted significant in PFS in sunitinib vs pazopanib patients (GG+CG>CC in sunitinib, CC>GG+CG in pazopanib; p<0,0001); VEGF A rs699947 resulted significant in PFS in sunitinib vs pazopanib patients (AA+AC>CC in sunitinib, CC>AA+AC in pazopanib; p<0,0001). OS showed no statistically significant difference.

**Conclusions:**

in our analysis patients with opposite polymorphisms of rs833061, rs2010963, rs699947 of VEGF A seems to have a better PFS if treated with either sunitinib or pazopanib. Our data seem to suggest that biology could have a role choosing first line treatment for mRCC patients.

**Methods:**

a retrospective analysis on 97 histologic samples of mRCC patients was conducted for VEGF-A, VEGF-C and VEGFR-1,2,3 single nucleotide polymorphisms (SNPs).

## INTRODUCTION

Since the introduction of multitarget tyrosine-kinase inhibitors (TKIs) the survival scenario for metastatic renal cell carcinoma patients radically changed. Based on efficacy results, sunitinib was widely used as a first-line treatment until 2010. The introduction of pazopanib, a novel multi target inhibitor, put on the market a new alternative first-line drug. These two drugs exploit their action through the inhibition of angiogenesis by molecular interaction with almost the same receptors (VEGFRs, PDGFR, and KIT) but with different affinity. Results from the COMPARZ study, the first phase III trial comparing sunitinib and pazopanib, has been recently published. This trial was designed to demonstrate a non-inferiority of these therapeutic options, in terms of efficacy and according to quality of life parameters. Although the study showed a non-inferiority in terms of PFS of pazopanib versus sunitinib, it also suggested that pazopanib might have a better tolerability [1]. On the one hand these two drugs can be then considered valuable options for first-line treatment. However, on the other hand, a possible choice based on quality of life profiles may result questionable. In fact the questionnaires used, the timing of test administration and the possible influence between the patient and the physician are the major downsides of the analysis.

Globally, the possibility of a wider choice for first-line treatment makes the research for predictive markers of response even more relevant to improve global clinical outcome. A more efficient selection would also allow avoiding unnecessary toxicities to potentially resistant patients.

Angiogenesis hyperactivation, as a compensatory response to hypoxia, is particularly important in RCC. RCC are, in fact, highly vascularized tumours and are frequently associated with mutations in *VHL* gene, a critical regulator of the hypoxic response, for the onset of the disease [2].

Single-nucleotide polymorphisms (SNPs) in the vascular endothelial growth factors (VEGF) and VEGF receptors (VEGFR) genes have been also correlated to tumour neoangiogenesis through different biological mechanisms.

Currently numerous evidence are contributing to correlate angiogenesis SNPs and global outcome in several diseases such as colorectal, breast and ovarian cancers, when treated with antiangiogenic therapy [3–7], but data in mRCC are lacking. In a study performed on blood samples and tumour tissue specimens, Kim et al. showed a statistically significant difference in patients with SNP −634 for sunitinib related hypertension [8]. Another study published by Garcia-Donas et al. correlated SNPs with response and toxicities in mRCC patients treated with sunitinib. Polymorphisms of VEGFR3 and CYP3A5*1 are proposed in this paper as potential markers of tolerability and response [9].

We previously reported how VEGF and VEGFRs SNPs are able to predict outcome in patients treated with sunitinib. VEGF A polymorphism rs833061, rs699947 and rs2010963 and VEGFR 3 rs6877011 seem to influence the outcome of patients with metastatic renal cell carcinoma treated with sunitinib [10].

Similarly the influence of various SNPs was assessed, in an unplanned analysis, by Xu et al. in patients enrolled in the pivotal trial about pazopanib. They evaluated in blood samples the correlation between SNPs of the angiogenic pathway, including some of VEGF, with survival and toxicity. In this study VEGFA _1498 CC genotype compared with the TT genotype conferred inferior PFS and RR (33% v 51%) [11].

Recently Marisi et al. showed how polymorphisms expression of VEGF was preserved between peripheral blood and formalin fixed paraffin embedded tisues (FFPE) in patients with colorectal cancer. They analysed 237 patients samples, peripheral blood was used for 153 patients, whereas only FFPE tumor tissue was available for 84 patients. All VEGF and eNOS polymorphisms, apart from VEGF −1154G>A, were comparable in peripheral blood and FFPE samples, suggesting that FFPE tissue is a valuable source of biological material on which the majority of molecular studies can be performed. [12].

The aim of the present study is to assess whether a difference in polymorphisms expression could be able to predict different outcomes in patients treated with sunitinib or pazopanib.

## RESULTS

The following SNPs met our selection criteria: VEGF-A: rs25648, rs10434, rs833061, rs699947, rs2010963, rs3025039; VEGF-C: rs4604006, rs7664413. VEGFR-1: rs664393, rs7993418; VEGFR-2: rs2071559, rs2305948, rs1870377, rs7667298; VEGFR-3: rs307822, rs307805, rs6877011 (Table 1).

**Table 1:** Chromosomal locations, positions, biological effects and minor allele frequencies in the study population of investigated gene SNPs

SNP ID	Gene	Chr	Chr. Position	Position in the gene/Effect	Codon exchange	aa. exchange	Minor allele frequencies
**rs10434**	VEGFA	6	43753212	3′UTR^(c)^	-	-	A - 40,43%
**rs2010963**	VEGFA	6	43738350	5′UTR^(d)^	-	-	C - 32,29%
**rs25648**	VEGFA	6	43738977	Syn^(a)^; ESE^(b)^	TCC ⇒ TCT	S [Ser] ⇒ S [Ser]	T - 17,02%
**rs3025039**	VEGFA	6	43752536	3′UTR^(c)^	-	-	T - 16,67%
**rs699947**	VEGFA	6	43736389	Prom^(e)^	-	-	C - 50,00%
**rs833061**	VEGFA	6	43737486	Prom^(e)^	-	-	T - 48,91%
**rs4604006**	VEGFC	4	177608775	Intronic	-	-	T - 19,79%
**rs7664413**	VEGFC	4	177608707	Intronic	-	-	T - 8,51%
**rs664393**	FLT1	13	29071001	3′UTR^(c)^	-	-	A - 12,77%
**rs7993418**	FLT1	13	28883061	Syn^(a)^; ESE^(b)^	TAC ⇒ TAT	Y [Tyr] ⇒ Y [Tyr]	G - 22,92%
**rs1870377**	KDR	4	55972974	Missense	CAA ⇒ CAT	Q [Gln] ⇒ H [His]	A - 18,18%
**rs2071559**	KDR	4	55992366	Init. Transcription	-	-	T - 45,83%
**rs2305948**	KDR	4	55979558	Missense	GTA ⇒ ATA	V [Val] ⇒ I [Ile]	T - 3,13%
**rs7667298**	KDR	4	55991731	5′UTR^(d)^	-	-	T - 48,96%
**rs307805**	FLT4	5	180077487	Prom^(e)^; TFBS^(g)^	-	-	A - 7,45%
**rs6877011**	FLT4	5	180029471	3′UTR^(c)^	-	-	G - 5,56%
**rs307822**	FLT4	5	180028717	3′UTR^(c)^	-	-	G - 12,77%

(a) Syn: Synonymous substitution

(b) ESE: Exon Splicing Enhancer

(c) 3′UTR: Untranslated Region 3′UTR

(d) 5′UTR: Untranslated Region 5′UTR

(e) Prom: Promoter region

(g) TFBS: Predicted Trascription Factor Binding Site.

All SNPs genotyped presented an overall call rate ≥ 90%.

We have evaluated concentration and purity index of each sample by UV spectrophotometry as the ratio absorbance 260/280 nm. All samples presented a purity index between 1.5 and 2.0.

The frequencies of the tested genotypes resulted comparable to those reported in Caucasians, with no significant deviation from the Hardy-Weinberg equilibrium.

Linkage disequilibrium was observed for the tumour genotypes rs833061, rs699947 and rs2010963 of VEGF A (p>0,0001).

Ninety-seven patients with histologically proven mRCC, receiving first-line sunitinib or pazopanib were available for our analysis: 60 males and 18 females in the sunitinib group; 11 males and 8 females in the pazopanib group. Median age at diagnosis of 65 years (range 47-85) in the general population with 64 years for sunitinib and 68 years for pazopanib (Table 2).

**Table 2:** Patients' characteristics

		Sunitinib	Pazopanib
**Number of patients**		78	19
**Gender**	Male	60 (77%)	11 (58%)
	Female	18 (33%)	8 (42%)
**Median age (range 47-84)**	>65	38 (49%)	10 (53%)
	<65	40 (51%)	9 (47%)
**Surgery**	Yes	64 (82%)	15 (79%)
	No	14 (18%)	4 (21%)
**Histology**	Clear cell	73 (93%)	17 (89%)
	Other	5 (7%)	2 (11%)
**ECOG**	0	57 (73%)	13 (68%)
	1	13 (17%)	4 (21%)
	2	8 (10%)	2 (11%)
**Stage at diagnosis** *(AJCC Cancer Staging Manual. 2010)*	I	4 (5%)	0 (0%)
	II	7 (9%)	1 (5%)
	III	40 (51%)	8 (42%)
	IV	27 (35%)	9 (47%)
**Best Response**	CR	1 (1%)	0 (0%)
	PR	9 (12%)	2 (10%)
	SD	23 (29%)	7 (37%)
	PD	45 (58%)	10 (53%)
**Risk classification (Heng score)**	Favourable	20 (26%)	5 (26%)
	Intermediate	44 (56%)	11 (57%)
	Poor	14 (18%)	3 (17%)

Seventy-five patients underwent renal surgery (77%, 64 sunitinib, 15 pazopanib), for twenty-two patients only core biopsies were available (23%, 14 sunitinib, 4 pazopanib). Thirty-six patients were metastatic at diagnosis (41%, 27 sunitinib, 9 pazopanib). Ninety patients had a clear cell renal cell carcinoma histology (74 sunitinib, 18 pazopanib), whether 7 patients presented undefined histology. All patients received sunitinib with standard schedule 50mg/die (4 weeks on/2 weeks off) or pazopanib at standard dose 800mg/die continuously as first-line treatment, dose reduction was applied in patients with grade 3 and 4 toxicities, as clinically indicated. Seventy-eight patients received sunitinib while 19 received pazopanib.

In the general population median PFS was 6,79 months, while median OS was 18,62 months.

No statistically significant differences were found according to treatment received for major patients characteristics (PS, tumour burden, grading, etc.).

Survival analysis was conducted dividing patients according to the administered drug and for each drug according to the polymorphism expression.

**Figure 1:**
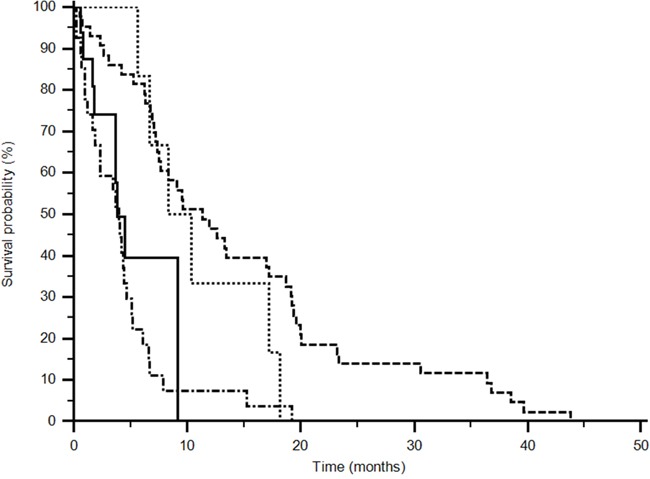
Progression Free Survival analysis of rs833061 (p<0,0001) (------ CC+CT sunitinib) (••••• TT pazopanib) (_____TT sunitinib) (−•−•−•− CC+CT pazopanib). Different polimorphisms expression confers a significant difference in outcome (p<0,0001; logrank test: Chi-square 30,0448).

Fifty-two patients (66%) expressed the CC+CT genotype of rs833061 while 26 (34%) expressed TT genotype in the sunitinib group, instead 13 (68%) CC+CT and 6 (32%) TT genotype in the pazopanib group. An advantage in median progression free survival was observed for patients expressing the CC+CT genotype in the sunitinib group (11,3 vs 4 months) and for patients with the TT genotype in the pazopanib group (9,3 vs 3,8 months) (p<0,0001; logrank test: Chi-square 30,0448) (Figure 1).

**Figure 2:**
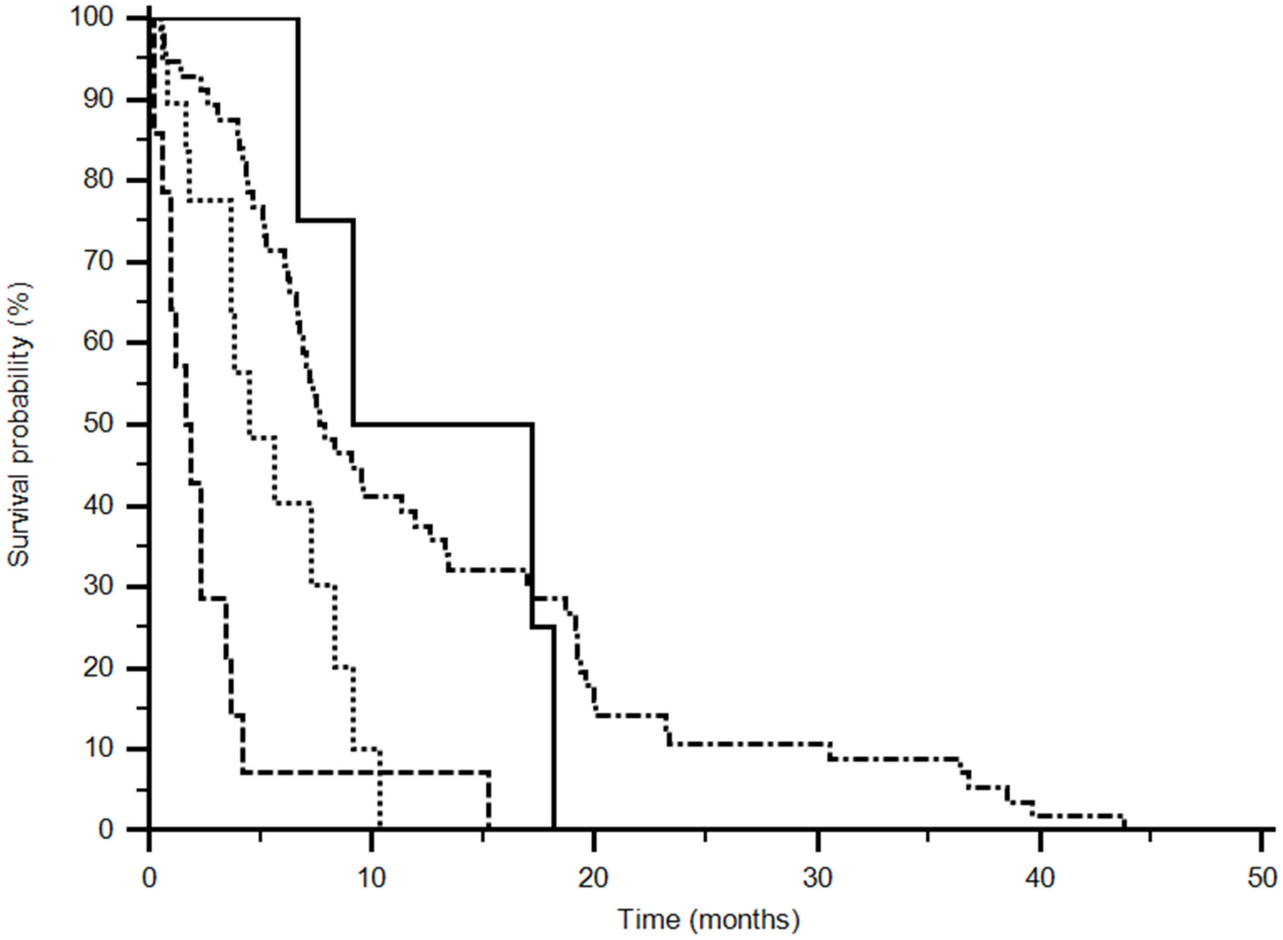
Progression Free Survival analysis of rs2010963 (p<0,0001) (------ CC sunitinib) (••••• GG+CG pazopanib) (_____CC pazopanib) (−•−•−•− GG+CG sunitinib). Different polimorphisms expression confers a significant difference in outcome (p<0,0001; logrank test: Chi-square 29,6518).

Fifty-nine (76%) patients expressed the GG+CG genotype and 19 (24%) the CC genotype in the sunitinib group for rs2010963, while 15 (79%) showed GG+CG genotype and 4 (21%) CC genotype in the pazopanib treated group. Median PFS was improved for the GG+CG genotype in the sunitinib group (7,7 vs 1,9 months), in the pazopanib group the CC genotype correlated with a better PFS (13,18 vs 4,48 months) (p<0,0001; logrank test: Chi-square 29,6518) (Figure 2).

**Figure 3:**
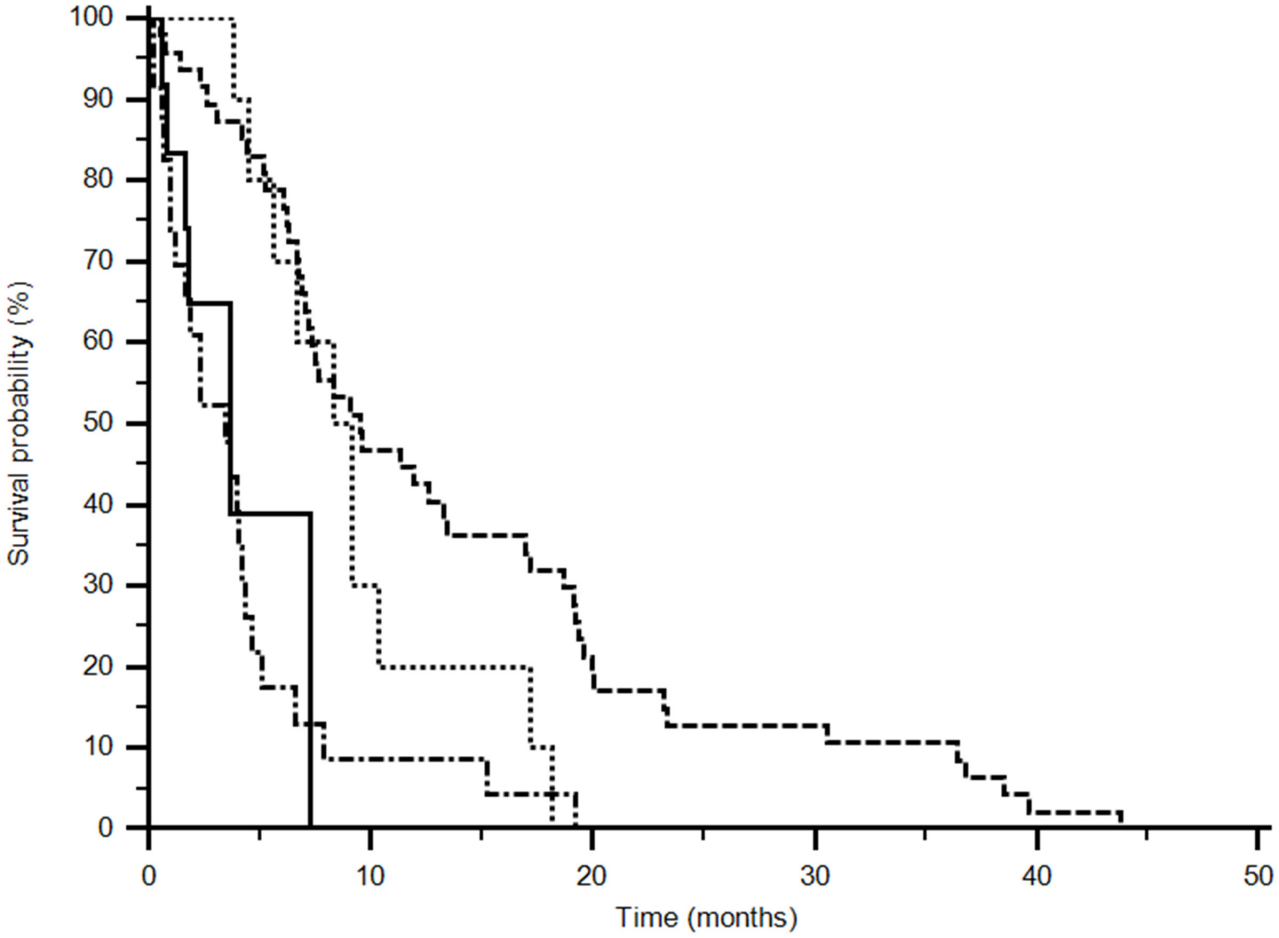
Progression Free Survival analysis of rs699947 (p<0,0001) (------ AA+AC sunitinib) (••••• CC pazopanib) (______ AA+AC pazopanib) (−•−•−•− CC sunitinib). Different polymorphisms expression confers a significant difference in outcome (p<0,0001; logrank test: Chi-square 35,6301).

Fifty-two patients (66%) showed the AA+AC genotype of rs699947 and CC genotype was expressed in 26 (34%) patients treated with sunitinib; in the pazopanib group 13 (68%) showed the AA+AC and 6 (32%) the CC genotype. The AA+AC genotype demonstrated an improved median PFS for the sunitinib group (9,5 vs 3,44) while CC genotype improved median PFS in the pazopanib group (9,1 vs 3,7 months) (p<0,0001; Logrank test: Chi-square 35,6301) (Figure 3) (Table 3).

**Table 3:** Polymorphisms results in univariate and multivariate analysis

Polymorphism	Genotype	N. of Patients		PFS (months)			OS
		SUN	PAZ	SUN	PAZ		
**VEGF A**							
**rs833061**	CC+CT	52 (66%)	13 (68%)	11,3	3,8		
	TT	26 (34%)	6 (32%)	4	9,3		
						p<0,0001	NR
**rs2010963**	GG+CG	59 (76%)	15 (79%)	7,7	4,48		
	CC	19 (24%)	4 (21%)	1,9	13,18		
						p<0,0001	NR
**rs699947**	AA+AC	52 (66%)	13 (68%)	9,5	3,7		
	CC	26 (34%)	6 (32%)	3,44	9,1		
						p<0,0001	NR

Overall survival analysis did not reach statistical significance possibly due to a shorter follow-up in the pazopanib group patients.

## DISCUSSION

The principal molecular target for TKIs used in mRCC treatment is the VEGF pathway via inhibition of the tyrosine kinase activity of VEGF receptors-1, 2 and 3. However, TKIs are unspecific and inhibit a broad spectrum of other receptor tyrosine kinases to varying degrees. Newer TKIs have greater target selectivity and affinity for VEGF receptors and data suggest that these agents may cause fewer and/or less severe off-target, non-VEGF related side effects [13, 14]. However, off-target side effects are still observed with the newer TKIs and, in the case of pazopanib, also novel toxicities such as an increased risk of hepatic toxicity are described [15].

The ability to inhibit the key targets such as VEGF receptors and PDGFR is proposed to account for the efficacy of TKIs. TKIs with high affinity for VEGF receptors might therefore be expected to be more effective than TKIs with a lower affinity. However, median progression-free survival (PFS) of approximately 11 months is achieved with both the high affinity, more-selective TKI pazopanib [16, 17] and the lower-affinity TKI sunitinib. Differences in trial design, patient numbers and the use of dose reduction (sunitinib drug exposure [area under the curve] correlates with efficacy: reduced doses lead to reduced overall efficacy) [18] may partially explain these observations. Another explanation supported by preclinical pharmacokinetic data suggests a critical threshold for VEGF and other receptor inhibition by TKIs beyond which no additional gain in overall clinical efficacy is achieved [19].

Combined analysis on our series according to the administered drug showed a better PFS for patients expressing CC+CT polymorphism of rs833061, GG+CG polymorphism of rs2010963, AA+AC polymorphism of rs699947 in sunitinib patients, while TT polymorphism of rs833061, CC polymorphism of rs2010963, CC polymorphism of rs699947 in pazopanib patients. rs833061 is located in the promoter region of the VEGF A gene on chromosome 6, as rs699947. Instead rs2010963 is located in the terminal 5′ UTR region of the VEGF A gene. We can hypothesize that different SNPs in different regions of the VEGF gene may influence circulating levels of VEGF and thus response to anti-VEGF therapies, but their exact extent of influence is still unknown.

These findings could explain how a certain constitutive variation in VEGF and VEGFR levels in tumour cells could exert a significant difference in outcome during antiangiogenic treatment, even though candidate gene studies exploring associations between VEGF polymorphisms and circulating VEGF levels have yielded controversial results. Eight studies have found significant associations with candidate polymorphisms (rs699947, rs1570360, rs833061, rs2010963, rs3025039, and 2549 18bp I/D) in the promoter, 5′, and 3′ untranslated regions of the VEGF gene [20–26]. However, several other studies did not identify any association with these and other VEGF SNPs. Using a hypothesis-free genome-wide approach, Debette et al. found associations with 140 SNPs. Of these, 68 SNPs are located on chromosome 6, approximately 150 kb downstream from the 3′ end of the VEGF gene, far from previously tested candidate SNPs [27]. However the real effect of SNPs in circulating or tumour tissues VEGF levels needs further studies in order to definitively associate a specific SNP to a specific effect on the corresponding growth factor or receptor.

Although we are unable to define the exact biological effect of a genotype on the protein expression, in our analysis we showed how a different pattern of expression of polymorphisms leads to a different outcome according to the first-line drug administered. To notice is the opposite expression of VEGF A polymorphisms in patients with better outcome with sunitinib or pazopanib. This finding is in accordance with our previous report on sunitinib patients and with the analysis conducted on patients enrolled in the pazopanib trial [10, 12]. On the contrary another study by Garcia-Donas didn't find a correlation between PFS, OS and rs2010963 and rs699947 [9].

Sunitinib and pazopanib showed no statistical differences in their efficacy in our analysis, as reported in clinical trials. Nevertheless, a difference was found according to the biological genotype of tumoural cells. Patients with a favourable polymorphism had a good PFS either with sunitinib or pazopanib, but notably, the favourable setting was the opposite for the two drugs. Our data seem to suggest that biology could have a role in the choice of first line treatment for mRCC patients. Our findings are preliminary and on a retrospective cohort of patients, they need to be validated in prospective studies and larger populations to be confirmed and enter the everyday clinical practice.

## MATERIALS AND METHODS

Patients receiving first-line treatment for histologically proven advanced renal cell carcinoma with either sunitinib (78 patients) or pazopanib (19 patients), treated between 2010 to 2014, were eligible for analysis at our Institution.

Ethical Committee of our Institution approved the study design.

Follow-up consisted of physical examination, a complete blood count, chest radiography and US of the abdomen or CT/MRI scanning every 8 weeks or as clinically indicated.

VEGF and VEGFR genotyping was performed on formalin-fixed paraffin-embedded tissue block (about 30 mg) of renal cell carcinoma samples in nephrectomy or core biopsies, taken from the neoplasm periphery.

Paraffin wax was removed with xylene and the samples were washed twice with 100% ethanol. DNA was isolated from the deparaffinised tissue using the RecoverAll™ Total Nucleic Acid Isolation Kit for FFPE Tissues (Applied Biosystems, Foster City, CA, USA), according to the manufacturer's instructions. DNA from each sample was then eluted in 120 μl of eluting solution.

Single nucleotide polymorphisms (SNPs) within each gene were selected using the Pupasuite software (http://bioinfo.cipf.es - version 2.0.0, bioinfo 2008), the CIPF (Centro de Investigacion Principe Felipe) Single Nucleotide Polymorphism database (dbSNP) generated by the National Centre for Biotechnology Information (http://www.ncbi.nlm.nih.gov/SNP) and by review of the medical literature, using the following criteria:

1) the polymorphism had some degree of likelihood to alter the structure or the expression of the gene in a biologically relevant manner (i.e. affecting ese sequences, 3′ UTR or promoter region);
2) the minor allele frequency was above 10% (with the only exception of rs2305948, rs6877011 and rs307822);
3) the genetic polymorphism was established and well-documented.

Further considerations drove the selection of SNPs for our study. A correlation between the presence of a specific allele on a polymorphic site and the expression of the respective protein has been previously documented for VEGF [28, 29]. SNPs in regulatory sequences, such as introns and 5′ and 3′ UTRs, have been shown to affect mRNA stability, processing efficiency, isoform expression and localization. Moreover, regulatory motif sequences within the 3′ UTR of mRNAs have been shown to affect the stability of the messenger and/or its translational efficiency. Thus, it can be argued that SNPs in these sequences may influence VEGF and VEGF-R gene expression. Also on these bases, we selected the SNPs known to affect VEGF and VEGF-R expression and those located in regulatory sequences, for which a putative role in protein regulation can be assumed.

Globally we assumed that selected SNPs had impact on protein expression and therefore on biological function.

Selected SNPs were as follows: six polymorphisms in the VEGFA gene (rs10434, G>A; rs2010963, G>C; rs25648, C>T; rs3025039, C>T; rs699947, A>C; rs833061, C>T), two in VEGFC (rs4604006, T>C; rs7664413, C>T), two in VEGF-R1 (FLT1) (rs664393, G>A; rs7993418, A>G), four in VEGF-R2 (KDR) (rs1870377, A>T; rs2071559, A>G; rs2305948, G>A; rs7667298, A>G) and three in VEGF-R3 (FLT4) (rs307805, A>G; rs6877011, C>G; rs307822, G>A). Chromosomal locations, positions and biological effects of investigated VEGF and VEGFR SNPs have been summarised in Table 1.

SNP genotyping was performed by TaqMan technology, using SNP genotyping products (Applied Biosystems, Foster City, CA). Polymerase chain reaction (PCR) was performed and genotypes were analysed on the 7300 Real-Time PCR System (Applied Biosystems, Foster City, CA) using an ABI Prism 7300 Sequence Detection System software (version 1.3.1, Applied Biosystems, Foster City, CA). Each reaction contained 0.2 μl of total genomic DNA. Laboratory personnel blinded to patient status performed Genotyping, and a random 10% of the samples were repeated to validate genotyping procedures.

All SNPs genotyped had to present an overall call rate of ≥ 90% to be included in our analysis, all samples resulted significant during the analysis and didn't need test repetition.

Primary aim of our study was PFS, OS was considered as secondary end-point.

In order to obtain a significant difference in the proportion of patients free of progression at 6 months according to genotyping and assuming a 6 months progression free survival (PFS) of 55% and ≥ 80% as a target, at least 85 patients were necessary with α=0.05 and β=0.05.

Statistical analysis was performed with the MedCalc software version 10.4.8 for Windows.

The association between categorical variables was estimated by Chi-square test.

Survival distribution was estimated by the Kaplan–Meier method (Kaplan and Meier, 1958).

A significant level of 0.05 was chosen to assess the statistical significance.

For statistical analysis, overall survival (OS) and progression free survival (PFS) were defined as the interval between the date of beginning of first-line treatment to death or last follow-up visit, and to clinical progression or death or last follow-up visit if not progressed.

All polymorphisms were examined for deviation from Hardy-Weinberg equilibrium using the Powermarker v. 3.25 package (www.statgen.ncsu.edu/powermarker).

Linkage Disequilibrium (LD) analysis was also performed using the Powermarker v. 3.25 package (www.statgen.ncsu.edu/powermarker). LD was estimated using r2, with r2=1 indicating complete LD and r2=0 indicating absent LD.
